# Organs at risk proximity in central lung stereotactic ablative radiotherapy: A comparison of four-dimensional computed tomography and magnetic resonance-guided breath-hold delivery techniques

**DOI:** 10.1016/j.phro.2025.100761

**Published:** 2025-04-02

**Authors:** Nicolas Giraud, Hilâl Tekatli, Famke L. Schneiders, John R. van Sornsen de Koste, Marco Marzo, Miguel A. Palacios, Suresh Senan

**Affiliations:** a Amsterdam UMC Location Vrije Universiteit Amsterdam, Department of Radiation Oncology, Boelelaan 1117, Amsterdam, the Netherlands; b Cancer Center Amsterdam, Treatment and Quality of Life, Amsterdam, the Netherlands; c UMC Utrecht, Department of Radiation Oncology, Utrecht, the Netherlands

**Keywords:** MR-guided radiotherapy, IGRT, SABR, Lung

## Abstract

Higher toxicity rates are associated with stereotactic ablative radiotherapy (SABR) to central lung tumors. Breath-hold (BH) magnetic resonance-guided SABR (MR-SABR) can reduce doses to organs at risk (OAR). We quantified the planning target volumes (PTV) to OAR distance in 45 lesions treated using MR-SABR and generated a corresponding four-dimensional computed tomography (4D-CT) based PTV (motion-encompassing internal target volume plus 5 mm). For lesions located ≦3 cm from airways, BH MR-SABR increased the median PTV distance to OAR by 3.7 mm. For lesions ≦3 cm from pericardium, median PTV-OAR separation increased by 2.0 mm with BH. These findings highlight the advantage of BH SABR for central lung tumors.

## 1 Introduction

The International Association for the Study of Lung Cancer (IASLC) has classified central tumors as ≤2 cm in all directions of any critical organs at risk (OAR), including the proximal bronchial tree (PBT), esophagus, heart, brachial plexus, major vessels, spinal cord, phrenic and recurrent laryngeal nerves [1]. Up to a third of patients with tumours in the proximity of central airways may experience haemorrhage, atelectasis, or pneumonitis after stereotactic ablative radiotherapy (SABR) [2], [3], [4], [5]. In the ‘Expanded HILUS’ study, patients who were at increased risk of grade 5 bleeding and other fatal toxicity had received a maximum dose to D0.001 cc of the mainstem bronchi and the intermediate bronchus [2]. Radiation doses to cardiac substructures have also been associated with cardiac events or overall survival in previous studies [6], [7].

Breath-hold (BH) SABR delivery allows for use of reduced planning target volumes (PTVs), and consequently lower doses to critical structures. Smaller PTV’s are achievable with magnetic resonance-guided lung SABR (MR-SABR) for central tumors, thanks to real-time tumor tracking and automatic beam gating delivery [8]. MR-SABR has also been performed during free-breathing (FB) [9]. The MR-SABR approach is more resource intensive in comparison to SABR on standard linacs, making it desirable to identify patients who are most likely to benefit from this approach. This information will also facilitate referrals for inclusion in prospective clinical trials now underway [NCT05354596; NCT04925583; NCT04917224] [10], [11].

To study the potential of BH SABR delivery, we retrospectively studied patients who had undergone breath-hold MR-SABR for central tumors. All tumor positions were classified according to criteria used in the HILUS study [12], and we studied the proximity of the proximal bronchial tree and heart with both MR-SABR and an internal target volume (ITV) based SABR using a corresponding planning four-dimensional computed tomography (4D-CT).

## 2 Material and Methods

### 2.1 Patient selection and data collection

An Ethics-approved institutional database of patients treated with MR-SABR between 2016–2022 was accessed to identify central tumors. The latter was defined according to the IASLC classification (2015), i.e. within 2 cm in all directions of any mediastinal critical structure, including the bronchial tree, esophagus, heart, brachial plexus, major vessels, spinal cord, phrenic nerve, and recurrent laryngeal nerve [1]. This study was approved by our Medical Ethics Review Committee (#2018.602, IRB00002991).

Eligible patients had to have pre-treatment planning 4D-CT scans available. 4D-CT scans were commonly performed in our previous MR-SABR workflow until 2018 [10], [13]. Thereafter 4D-CT scans were available in about 50 % of cases, mainly patients initially worked up for CT-guided SABR, but in whom proximity of OAR’s led to referral for MR-SABR.

For this study, eligible patients had their tumor location reclassified based on the three-dimensional (3D) distance from gross tumor volume (GTV) edge to the bronchi, as was reported for the HILUS trial [12]. Group A comprised GTV locations < 1 cm from the trachea or main bronchi on a BH CT scan. GTV’s ≥ 1 cm from the trachea or main bronchi, but < 1 cm from the intermediate bronchus or lobar bronchi were defined as group B. Central tumors located ≥ 1 cm from the PBT, but < 2 cm from mediastinum, were classified as 'group C'.

### 2.2 MR-guided lung SABR workflow and 4D-CT acquisition

Our MR-guided SABR workflow for lung lesions has been previously described [10], [14]. Briefly, a volumetric MR using a TrueFISP pulse sequence acquisition was performed for simulation on the MRIdian system (ViewRay Inc., Mountain View). Tumor motion was characterized on sagittal, coronal, and axial planes, and GTV delineated on the most representative sagittal plane, followed by a visual assessment of automatic tumor contour tracking to establish feasibility of tracking on MR simulation. Next, a 3D BH CT scan was performed for dose calculation. Treatment plans were generated with the MRIdian treatment planning system, and typically consisted of step-and-shoot intensity-modulated radiotherapy (IMRT) beams, with daily re-optimization based on the GTV and OAR positions on a new BH MR scan. Most BH scans for MR-SABR were performed during light inspiration for comfort and compliance purposes, and occasionally in expiration. An expiratory phase was selected if either patient tolerance or when the target volume position suggested a higher gating efficiency was possible in expiration.

Clinicians could choose the on-table reoptimized plan or the baseline plan. SABR delivery was then performed during repeated BH assisted by a real-time patient visualized feedback system of actual GTV positions and the gating window, with an isotropic tracking boundary of 3 mm around the BH GTV. No respiratory-limiting interventions such as compression devices were used.

### 2.3 Analysis of study target volumes

All imaging, structure sets, and dose plans were imported in Velocity v4.1 (Varian Medical Systems, Inc.) for analysis. The breath-hold planning GTV and PTV (PTV_BH_) used for clinical MR-SABR plans were extracted from DICOM-RT data. On the MRIdian MR-linac, a margin of 5 mm was added to create the PTV (PTV_BH_) from gross tumor volumes (GTV) on the BH MRI scan in shallow inspiration. For patients treated on the earlier MRIdian Cobalt-60 system, a 3 mm PTV margin was used. For the present study, the PTV_BH_ for Cobalt-60 plans were re-generated using a 5 mm margin around the GTV.

For this analysis, a motion-encompassing ITV was created using the corresponding 4D-CT planning scan acquired in FB. All study contours were reviewed by a second physician. An ITV-PTV margin of 5 mm in all directions was applied to create the PTV_FB_.

### 2.4 OAR delineation and assessment of proximity to target volumes

Lumens of the trachea and main stem bronchi were contoured on the baseline MR scan in a fixed contrast setting of 150 arbitrary units, and in the lung window-level settings (level −400, width 1200 HU) on the average intensity projection (Ave-IP) of the 4DCT scan. Contoured luminal structures were expanded with an isotropic 3 mm margin to derive airway wall. The heart was contoured as the full pericardium up to the pulmonary trunk and right pulmonary artery separation on baseline MR scans, and contours subsequently rigidly propagated to the BH planning CT used for MR-SABR [15]. The baseline planning CT was used to propagate heart contours to the Ave-IP of the pre-treatment 4D-CT scan using structure-guided deformable image registration. Finally, corresponding heart contours were reviewed on the Ave-IP scan in mediastinum window-level settings (level 40, width 350 HU), and manually edited if necessary. All OAR delineations were reviewed by a second physician.

**Fig. 1:**
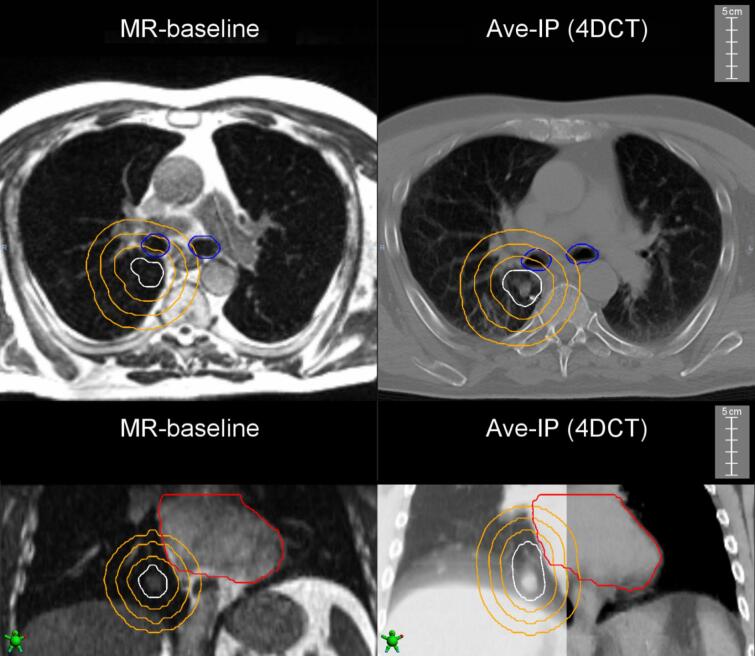
Examples of differences between PTV_BH_ (left) and PTV_FB_ (right) for a tumor close to the airways (upper panel), and a second tumor close to the heart (lower panel). The PTV is shown in white. The orange rings indicate the 1-, 2-, and 3-cm expansion of the PTV structure. (For interpretation of the references to colour in this figure legend, the reader is referred to the web version of this article.)

The proximity of the heart and major airways to the target volumes was quantified as (1) the minimum distance in 3D (in mm) of the OAR to the peripheral edge of the PTV (closest point belonging to the PTV contour from the OAR) and (2) the overlapping volume (in cc) of the OAR with the 1, 2 and 3 cm ring structures around the PTV (Fig. 1). All 3D distances and volumes were derived using the tools in the Velocity software.

### 2.5 Statistical and dosimetric analysis

Excel (Microsoft, Redmond, USA) was used to sort and tabulate all numerical data. Descriptive statistics and two-tailed *t*-test were used to compare paired and unpaired data sets respectively. A p-value threshold of 0.05 was used to indicate statistical significance. All planned baseline doses were extracted from the ViewRay planning system and converted to 2 Gy per fraction equivalent doses (EQD_2_) using an α/β ratio of 3.

## 3 Results

### 3.1 Patient characteristics

Between 2016–2022, 193 lung lesions were treated using MR-guided lung SABR, of which 119 fulfilled the IASLC criteria for central tumors. 4D-CT scans were available in 43 patients treated for 45 lesions (Table 1). Tumor locations on the BH MR scan were classified as HILUS group A in 7 % of lesions, group B in 13 %, and group C in 80 %. Most tumors (56 %) were in the lower lobes.

**Table 1:** Characteristics of 45 central lung lesions treated in 43 eligible patients.

Characteristics	N (%) or median (range)
Male gender	26 (60 %)
Age (years)	69 (35–91)
Fractionation scheme	
12 × 5 Gy	1 (2.2 %)
3 × 18 Gy	3 (6.7 %)
5 × 11 Gy	16 (35.6 %)
5 × 8 Gy	1 (2.2 %)
8 × 7.5 Gy	24 (53.3 %)
Tumor location	
Right lung	24 (53.3 %)
Upper lobe	8 (17.8 %)
Middle lobe	2 (4.4 %)
Lower lobe	14 (31.1 %)
Left lung	21 (46.7 %)
Upper lobe	10 (22.2 %)
Lower lobe	11 (24.4 %)
GTV location	
Group A	3 (6.7 %)
Group B	6 (13.3 %)
Group C	36 (80.0 %)
GTV_MRI_ size (cc)	8.6 (0.6–68.2)
PTV_BH_ size (cc)	25.6 (4.5–126.8)
PTV_FB_ size (cc)	33.3 (8.2–195.0)

Abbreviations: GTV: gross tumor volume; PTV: planning target volume; MRI: magnetic resonance imaging; BH: breath-hold; FB: free-breathing.

Group A = gross tumor volumes (GTV) located ≤ 1 cm from the trachea or main bronchi. Group B: GTV located > 1 cm from the trachea or main bronchi, but ≤ 1 cm from the bronchus intermedius or lobar bronchi. Group C = GTV located > 1 cm from the proximal bronchial tree, but ≤ 2 cm from the mediastinum.

### 3.2 Target volumes

Median GTV for all treated lesions was 8.6 cc (range, 0.6–68.2), and median ITV was 12.3 cc (range 1.9–116.5), corresponding to a median ITV/GTV ratio of 1.4 (range 1.1–5.1). This ratio was significantly higher for lower lobe lesions when compared to other lobes (medians of 1.2 versus 1.8, p = 0.001). Median PTV_BH_ was 25.6 cc (range 4.5–126.8) and this was 33.3 cc (range 8.2–195.0) for PTV_FB_, with a median PTV_FB_/PTV_BH_ ratio of 1.3 (range 1.1–3.3).

### 3.3 Planned doses from baseline MR treatment plans

Most lesions were treated using 8 fractions of 7.5 Gy (53 %). Median heart EQD2 D0.03 cc, D0.5 cc and D1.0 cc were 70.5 Gy (range 1.3–163.9), 64.0 Gy (range 1.2–152.1) and 57.7 Gy (range 1.1–146.6). For the major airways, median EQD2 D0.03 cc, D0.5 cc and D1.0 cc were 22.9 Gy (range 1.2–100.6), 13.2 Gy (range 0.9–94.1) and 8.6 Gy (range 0.9–89.4).

### 3.4 Proximity of target volumes to the major airways

Median distances from the major airways to the PTV_BH_ and PTV_FB_ were 36.0 mm (range 0.0–113.2) and 34.5 mm (range 0.0–113.0), respectively. The median increase in distance was 3.8 mm (range −0.09 to +15.9) with PTV_BH_ (p < 0.001) (Supplementary Fig. S1, top panel). Increased distance to major airways was significantly greater in patients with a tumor location in the lower lobes (medians of 6.2 versus 1.9 mm, p = 0.002).

A subgroup of 20 tumors located within 3 cm of the major airways were further analyzed based on the FB Ave-IP scan analysis. Median overlap of PTV_BH_ with the 1, 2 and 3 cm ring structures was 0.0 cc (range 0.0–4.9), 0.0 cc (range 0.0–20.0), and 1.0 cc (range 0.05–40.5), respectively. Corresponding volumes for the PTV_FB_ were 0.0 cc (range 0.0–12.3), 0.0 cc (range 0.0–30.9), and 2.2 cc (range 0.0–50.1), respectively (p > 0.05 for all rings). Use of PTV_BH_ allowed a median increase in distance to the proximal airways of 3.7 mm (range, 0.0–9.9) in this subgroup, also predominant for lesions of the lower lobes (medians of 5.5 mm for lower lobe tumors, versus 2.8 mm for the other lesions).

### 3.5 Proximity of target volumes to the heart

Median distance of the pericardium to the edge of the PTV_BH_ was 15.8 mm (range 0.0–82.6), and 10.7 mm for the PTV_FB_ (range 0.0–82.9) (p < 0.001). With PTV_BH_ a median increase in distance of 2.2 mm (range −2.8 to + 10.9; p < 0.001) was observed (Supplementary Fig. S1, bottom panel).

A total of 38 lesions were located within 3 cm from the heart contour based on the FB Ave-IP scan analysis. For the PTV_BH_, median overlap of the heart with the 1, 2 and 3 cm ring structures was 0.0 cc (range 0.0–29.6), 7.3 cc (0.0–78.9), and 30.5 cc (0.0–148.2), respectively. These values were 0.2 cc (range 0.0–43.8), 9.5 cc (0.0–101.3), and 38.9 cc (range 0.3–184.6) for the PTV_FB_. A significant lower overlapping volume was observed for PTV_BH_ for all ring structures (p < 0.001 for all). In terms of distance, PTV_BH_ allowed a median distance increase to the heart of 2.0 mm (range −2.1 to 10.9) in this population.

## 4 Discussion

Of all patients undergoing MR-SABR at our institution, nearly 62 % had tumors classified as being central in accordance with IASLC criteria, a finding that reflects the awareness of increased toxicity after SABR delivered to lung tumors at this location. The main findings of this study in a subset of 45 tumors treated using MR-SABR and in whom 4DCT scans were available was that a significant increase in distance between tumor and OAR's was obtained with BH than when using an ITV generated by conventional 4DCT planning scans. Specifically, in 20 tumors located <3 cm from main airways, BH led to a median increase in distance between the PTV and main airways by 3.7 mm (range 0–9.9), with a greater benefit for lower lobe lesions. In 38 lesions located <3 cm from the pericardium, BH led to a median increase in distance to the heart of 2.0 mm (range −2.1 to 10.9). Given the steep dose fall-off with MR-SABR delivery [10], these findings suggests that BH procedure is particularly beneficial for this group of central tumors. As only 20 % of treated tumors were classified as being in the HILUS groups A and B, i.e. located ≤1 cm from the PBT, our findings highlight the importance of ensuring that such details are reported in comparisons between trials treating all central tumors.

Growing data supports a role for minimizing radiated volumes of major airways as an approach for reducing clinical and radiological airway toxicity [2], [3], [12], [16]. The expanded HILUS trial reported a 9 % incidence of fatal lung hemorrhage in a series of 230 lung SABR patients with a tumor located within 2 cm from the PBT [2]. Almost 95 % of patients experiencing fatal lung hemorrhage had a tumor located within 1 cm from the major airways. Radiation doses to cardiac substructures have been correlated with non-cancer death in early-stage lung cancer [17].

We acknowledge some limitations of our work, including the lack of reliable constraints for central airway organs at risk (OAR) for lung SABR [18]. As planning techniques and dose-distributions can be further optimized in research studies, we restricted our study to only a geometric analysis, using geometric parameters as a surrogate for dosimetric and clinical outcomes. Dosimetric comparisons for central and ultracentral lesions are challenging as they often rely on trade-offs between imperfect target coverage and reasonable OAR doses. Single 4D-CT volumetric modulated arc therapy (VMAT) plans may also not be free from dosimetrist bias. Furthermore, use of an ITV-based PTV and an average intensity projection of an initial planning 4D-CT scan may not reliably reflect tumor motion during treatment [19], [20]. Heart contours may also be subject to interobserver bias, although we found reproducible heart volumes across different imaging techniques (data not shown).

In conclusion, use of geometric distance as a surrogate for both dosimetric and clinical outcomes, breath-hold MR-guided lung SABR could improve the therapeutic ratio in tumors located <3 cm from the PBT and heart by increasing the distance between the PTV and these OARs. Such patients are good candidates for ongoing prospective trials evaluating the use of MR-SABR, which are needed to prove its potential benefits in clinical outcomes such as local control and treatment-related mortality.

## Declaration of competing interest

The authors declare the following financial interests/personal relationships which may be considered as potential competing interests: S.S. and F.S. received institutional research grants from ViewRay Inc.
