# Supplementary material for: Organs at risk proximity in central lung stereotactic ablative radiotherapy: A comparison of four-dimensional computed tomography and magnetic resonance-guided breath-hold delivery techniques
Source: Phys Imaging Radiat Oncol. 2025 Apr 2;34:100761. doi: 10.1016/j.phro.2025.100761 (PMC12002652; doi:10.1016/j.phro.2025.100761)

Supplementary data

Supplementary Figure S1: Observed difference in minimum distances from planning target volumes (PTV) to major airways (blue bars) and to the heart (red bars) between free-breathing (PTV_{FB}) and breath-hold (PTV_{BH}). At Y-axis, positive numbers indicate the distance to the major airways and heart was larger for PTV_{BH}.

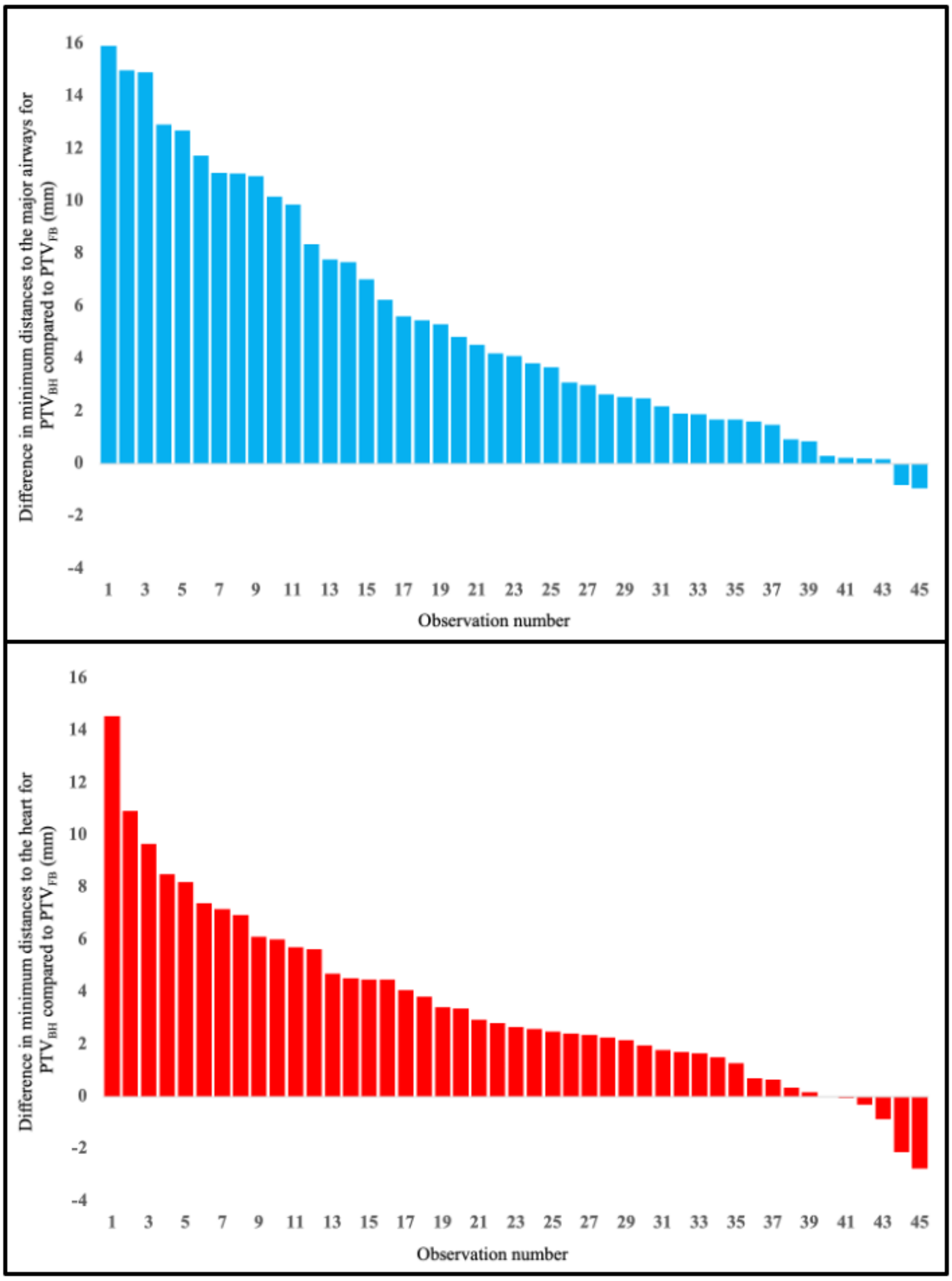

Supplement: Supplementary Data 1 [file mmc1.pdf]
